# A Dramatic Response to Toripalimab With Chemotherapy and Antiangiogenic Agent Followed by Surgery in a Stage IIIB Lung Adenocarcinoma Patient With an Uncommon *EGFR* Mutation: A Case Report

**DOI:** 10.3389/fonc.2022.915628

**Published:** 2022-07-11

**Authors:** Pengda Zhai, Xueliang Niu, Kai Zheng

**Affiliations:** ^1^ Department of Cardiothoracic Surgery, Tianjin Medical University General Hospital, Tianjin, China; ^2^ Department of Medical Affairs, Shanghai Junshi Biosciences Co., Ltd., Shanghai, China

**Keywords:** lung cancer, induction therapy, high PD-L1 expression, uncommon epidermal growth factor receptor mutation, toripalimab

## Abstract

Lung cancer patients with high programmed cell death-ligand 1 (PD-L1) expression in tumor cells and *epidermal growth factor receptor* (*EGFR*) mutations are rare, but there is no clinical standard for which treatment such patients should receive. Here, we report a 52-year-old male smoker who was diagnosed with stage IIIB lung adenocarcinoma. A rare *EGFR* G719A mutation was detected in the lymph node samples by next-generation sequencing (NGS), and a high PD-L1 expression was found by immunohistochemistry (IHC). After 10 cycles of induction therapy (toripalimab plus pemetrexed plus nedaplatin plus apatinib), surgery was successfully performed, followed by 2 cycles of consolidation therapy (toripalimab plus pemetrexed) and 4 cycles of maintenance therapy (toripalimab). A progression-free survival (PFS) of 7 months was achieved. In this case, we showed that the programmed cell death protein 1 (PD-1) inhibitor toripalimab plus chemotherapy and apatinib was effective and tolerable in a locally advanced *EGFR*-mutant non-small cell lung cancer (NSCLC) patient with a positive PD-L1 expression.

## Introduction

Approximately 80%–85% of lung cancers are non-small cell lung cancer (NSCLC) (1). Immune checkpoint inhibitors (ICIs) have demonstrated efficacy in the treatment of advanced NSCLC. Recently, a number of phase I–III clinical trials have provided new evidence for the use of ICIs in the neoadjuvant treatment of resectable (stage I–IIIA) or potentially resectable (stage IIIB) NSCLC. In resectable NSCLC, neoadjuvant treatment with nivolumab (2) and atezolizumab (3) showed a major pathologic response (MPR) rate of 45% and 21%, respectively, with tolerable toxicity and no increase in surgical mortality. In the CheckMate 816 study, neoadjuvant treatment of resectable NSCLC patients with nivolumab combined with chemotherapy was able to significantly prolong event-free survival and increase the complete pathological response rate compared with those with chemotherapy alone (4). However, neoadjuvant clinical data are still limited in potentially resectable NSCLC. Here, we report a man with locally advanced pulmonary adenocarcinoma (stage IIIb) with high programmed cell death-ligand 1 (PD-L1) expression an uncommon *epidermal growth factor receptor* (*EGFR*) mutation who was successfully treated with surgical resection following toripalimab, pemetrexed, nedaplatin, and apatinib.

## Case Presentation

**Figure 1:**
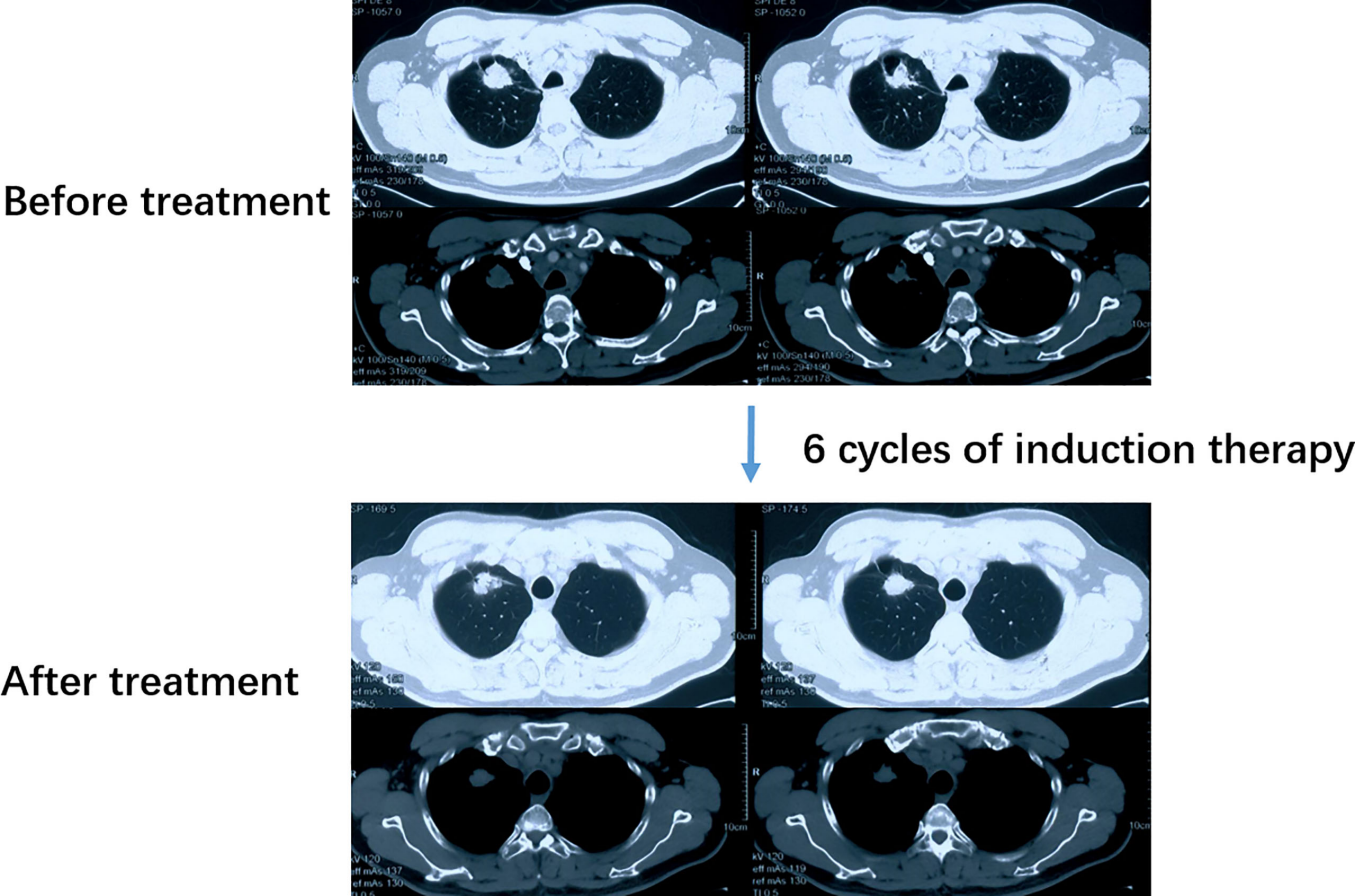
CT scan of the patient before and after induction therapy showed the tumor response. CT, Computed tomography.

**Figure 2:**
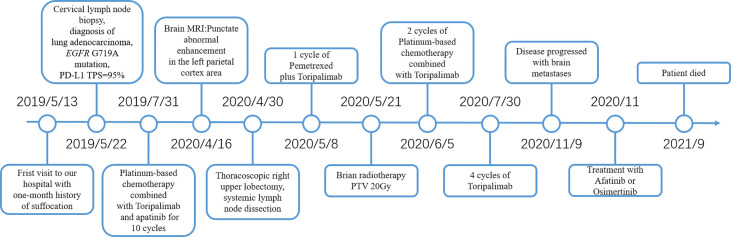
Timeline of diagnosis and treatment in this case. PD-L1, Programmed cell death-ligand 1; TPS, Tumor Proportion Score; MRI, Magnetic resonance imaging; PTV, Planning target volume.

A 52-year-old male smoker presented with 1-month history of chest distress in May 2019. Systemic evaluation including brain magnetic resonance imaging (MRI), whole-body bone scan, and chest contrast-enhanced computed tomography (CT) scan revealed a soft tissue mass on the upper lobe of the right lung (3.9 cm × 3.0 cm) and multiple enlarged lymph nodes in both supraclavicular fossa and mediastinum (**Figure 1**). He was diagnosed with stage IIIb lung cancer (cT_2_N_3_M_0_). Histopathology review of the right supraclavicular lymph node biopsy sample suggested lung adenocarcinoma. Notably, lymph node biopsy specimens tested by Dako 22C3 PD-L1 immunohistochemistry (IHC) showed 95% PD-L1 expression [Tumor Proportion Score (TPS)]. Molecular testing of lymph node biopsy samples using next-generation sequencing (NGS; 3DMed, Shanghai, People’s Republic of China) assays containing 733 cancer-related genes confirmed the presence of the *EGFR* G719A mutation with a mutant allele fraction (MAF) of 32.13% (**Table 1**), and tumor mutational burden (TMB) was calculated (4.03 mutations/Mb). Given the high PD-L1 expression of the biopsy sample and the fact that the patient was 52 years old, was in good physical condition at the time of initial treatment, and may tolerate antiangiogenic drugs well, the patient was given pemetrexed, nedaplatin, toripalimab (240 mg), and apatinib (250 mg) for the purpose of tumor downstaging as soon as possible for surgery. After six cycles of induction therapy, the patient achieved a partial response (PR) based on the Response Evaluation Criteria In Solid Tumors (RECIST) 1.1 (**Figure 1**) and the driver gene mutation of the blood sample by NGS testing had no change (**Table 1**). After eight cycles of treatment, the metastasized supraclavicular lymph nodes were no longer palpable. The mass in the right upper lobe was stable at 2.3 cm × 2.2 cm in size. During the treatment, the patient developed grade 1–2 rashes and grade 1 fever. The adverse events were tolerable and well controlled. No grade ≥3 toxicities were observed. Radical surgery for lung cancer was planned, but it was delayed by the Spring Festival, the coronavirus disease 2019 (COVID 19), and the patient’s own reasons. Finally, the induction therapy was performed for 10 cycles; CT showed that the mass in the right upper lobe became larger, with a maximum diameter at 2.3 cm × 3.0 cm, and punctate oligometastases in the brain were found. After multidisciplinary team (MDT) discussion, surgery was performed after 10 cycles of induction therapy on April 30, 2020. The surgical procedure includes thoracoscopic right upper lobectomy, systematic lymph node dissection, complex thoracic adhesiolysis, and closed drainage. One week after surgery, the patient received toripalimab 240 mg and pemetrexed 1 g, along with head radiotherapy (PTV20 Gy) 3 weeks after surgery. Pathology findings showed that the mass is 2.8 cm × 2.2 cm × 1.5 cm in size, and the residual tumor cells account for about 25%. A large number of CD8 cell infiltration were observed in the tumor area. NGS test of the tumor tissue sample detected the *EGFR* G719A mutation again (**Table 1**). After the radiotherapy, the patient received two cycles of toripalimab plus pemetrexed plus nedaplatin consolidation therapy and toripalimab maintenance therapy for 4 cycles. A progression-free survival (PFS) of 7 months was achieved. Unfortunately, the disease continued to progress with brain metastases. Even if we had changed to second-line treatment consisting of afatinib (30 mg/day) with a PFS of 7 months and third-line treatment consisting of osimertinib (double dosage: 160 mg/day) with a PFS of 2 months, eventually, we failed to prevent the progression of the brain metastases. The patient died in September 2021 (**Figure 2**).

**Table 1:** NGS-detected alterations (snv and indel) in the lymph node, plasma cfDNA, and tumor tissue.

Gene Symbol	cHGVS	pHGVS	Tumor_var_freq (Lymph node)	Tumor_var_freq (Plasma cfDNA)	Tumor_var_freq (Tumor tissue)
**BRCA2**	c.8888C>T	p.S2963L	5.41%	–	–
**EGFR**	**c.2156G>C**	**p.G719A**	**32.13%**	**0.11%**	**17.67%**
**FLT1**	c.3576C>G	p.D1192E	6.05%	–	–
**LRP1**	c.2062C>T	p.H688Y	7.84%	–	–
**MAGI2**	c.2371_2379del	p.R791_L793del	5.88%	–	–
**TP53**	c.559_559+1delinsTT	–	21.91%	–	11.76%
**TP53**	c.589G>C	p.V197L	**–**	–	3.49%

snv, single nucleotide variants; indel, insertion/deletion; cfDNA, cell free DNA; cHGVS, nucleic acid Human Genome Variation Society; pHGVS, protein Human Genome Variation Society; tumor_var_freq, tumor variation frequency; Bold text, driver gene mutations.

### Patient Perspective

“I felt very good during the treatment, and my appetite was significantly improved. The adverse events during treatment were slight and well controlled. Four months after surgery, I felt like a healthy normal person. I ran two marathons and did 90 kilometers of desert hiking.”

## Discussion

Due to heterogeneity, a single treatment concept cannot meet the clinical needs of all patients with stage III NSCLC. Neoadjuvant immunotherapy with anti-programmed cell death protein 1 (PD-1)/PD-L1 antibodies has recently yielded very encouraging results, providing more treatment options for patients with stage III NSCLC (2, 5, 6). Our patient was treated with induction immunotherapy using pemetrexed, nedaplatin, apatinib, and toripalimab for 10 cycles before surgery, and a PFS of 7 months was achieved.

In addition to the clinical phenotype, molecular characterization of tumor tissue should also be considered. At present, PD-L1 expression in tumor tissues can predict the therapeutic response to immunotherapy to some extent (7, 8). Our patient had a high level of PD-L1 expression in the tumor tissue (95%), which suggests that he may benefit from immunotherapy. However, more evidence is needed for PD-L1 expression as a biomarker to predict the efficacy of neoadjuvant immunotherapy for lung cancer.

The *EGFR* G719A mutation of our patient is another thing worth noting. It has been suggested that PD-1/PD-L1 inhibitors are less effective in patients with *EGFR* mutations than those with wild-type *EGFR (* 9, 10). Hence, the National Comprehensive Cancer Network (NCCN) guidelines recommend *EGFR* tyrosine kinase inhibitor (TKI) treatment for advanced NSCLC patients with *EGFR* mutations. However, the clinical evidence for targeted therapy in patients with uncommon *EGFR* mutations is still limited, and it is difficult to guide the choice of treatment options. In the LUX-Lung 2, LUX-Lung 3, and LUX-Lung 6 *post-hoc* analysis study (11), treatment with afatinib resulted in better PFS but worse overall survival (OS) than chemotherapy in patients with the G719X mutation alone (n = 8) and in combination with other mutations (n = 10). Therefore, other alternative treatment options need to be investigated. On the other hand, several reports have shown that some *EGFR* molecular subgroups might also benefit from PD-1/PD-L1 inhibitors. A pilot study showed a more significant PFS benefit with nivolumab in patients with uncommon *EGFR* mutations compared to that of patients with common *EGFR* mutations (12). Interestingly, small cohort studies have shown that ICIs (mainly second-line or later treatment) have better efficacy in patients with uncommon *EGFR* mutations compared to that of patients with common *EGFR* mutations (13, 14). As reported by Mazieres et al. (14), the uncommon *EGFR* mutation patients demonstrated relatively higher median PFS of 2.8 months (1.8 months for exon 19 and 2.5 months for exon 21, P < 0.001). The results of their study also suggested that PD-L1 positivity was significantly correlated with a longer PFS (14). Notably, a study found that patients with uncommon *EGFR* mutations had significantly increased PD-L1 expression compared with 19del/L858R mutations (49.0% vs. 12.2%) (15). Consistent with the literature, Dudnik et al. (16) also found that PD-L1-positive expression was 32% in rare target-driven cases. Taken together, these findings suggest that uncommon *EGFR* mutation subtypes tend to have high levels of PD-L1 expression and may benefit from immunotherapy. In our case, the patient carried the uncommon *EGFR* G719A mutation. Meanwhile, the patient’s PD-L1 expression was 95%. Thus, a combination of pemetrexed, nedaplatin, toripalimab, and apatinib was chosen. This new treatment strategy has achieved satisfactory results and may provide new therapeutic strategies for patients with stage IIIB NSCLC with uncommon *EGFR* mutations and positive PD-L1 expression. However, the findings need to be verified by further studies.

Dynamic monitoring of *EGFR* mutations *via* plasma cell-free DNA (cfDNA) could identify patients who might benefit from EGFR-TKIs and predict resistance to EGFR-TKIs (17). It is well known that plasma cfDNA is a good substitute for genetic testing in extracranial tumor tissue (18). However, evidence supporting the use of plasma cfDNA as a tissue surrogate for brain tumors is limited. Plasma cfDNA may not accurately provide genetic information of brain and meningeal lesions due to the presence of the blood–brain barrier. Cerebrospinal fluid (CSF) cfDNA may be more sensitive and specific than plasma cfDNA (19). So, we believe that further development of NGS technology is needed in order to predict the emergence of brain metastasis by *EGFR* mutations in plasma. In our case, before initiation of chemo-ICI-antiangiogenic treatment, the NGS testing was performed in lymph node but not in plasma. In addition, NGS testing was performed in plasma after six cycles of induction therapy and in surgery tumor tissue sample, and the results showed no change in the driver gene mutations. Possibly, more dynamic monitoring of plasma cfDNA gene mutation changes may be able to provide more useful prognostic information.

There are limitations in this case. First, there is no effective treatment regimen for our patient with brain metastases during consolidation therapy. This requires more clinical research to assist physicians in making optimal treatment options. Secondly, from our single case, we cannot make a conclusion that ICIs plus chemotherapy plus antiangiogenic agents can be widely used for induction therapy in other potentially resectable NSCLC cases with *EGFR* mutations. This requires validation in large cohort clinical trials.

## Conclusion

In conclusion, our case shows the successful treatment of an initially inoperable patient with *EGFR* G719A-mutated IIIB (cT2N3M0) NSCLC who became operable after the treatment with chemotherapy and apatinib with toripalimab. After surgery, consolidation therapy and toripalimab maintenance therapy was carried out, and a PFS of 7 months was achieved. In brief, high PD-L1 expression may be a predictive biomarker for the selection of PD-1 inhibitor for the treatment of uncommon *EGFR*-mutated NSCLC, and further optimization of therapeutic strategies warrants more investigation.

## Data Availability Statement

The original contributions presented in the study are included in the article/supplementary material. Further inquiries can be directed to the corresponding author.

## Ethics Statement

Written informed consent was obtained from the individual(s) for the publication of any potentially identifiable images or data included in this article.

## Author Contributions

KZ had the idea for the article and provided the final approval of the version to be published. PZ and XN performed the literature search, data analysis and drafted the manuscript. KZ was involved in revising the manuscript critically for important scientific content. All authors listed have made a substantial, direct, and intellectual contribution to the work and approved it for publication.

## Conflict of Interest

XN is employee of Shanghai Junshi Biosciences Co., Ltd.

The remaining authors declare that the research was conducted in the absence of any commercial or financial relationships that could be construed as a potential conflict of interest.

## Publisher’s Note

All claims expressed in this article are solely those of the authors and do not necessarily represent those of their affiliated organizations, or those of the publisher, the editors and the reviewers. Any product that may be evaluated in this article, or claim that may be made by its manufacturer, is not guaranteed or endorsed by the publisher.
